# Relationship between BMI and prognosis of chronic heart failure outpatients in Vietnam: a single-center study

**DOI:** 10.3389/fnut.2023.1251601

**Published:** 2023-11-30

**Authors:** Hoai Thi Thu Nguyen, Thuong Thi Thu Ha, Hieu Ba Tran, Dung Viet Nguyen, Hung Manh Pham, Phuong Minh Tran, Tuan Minh Pham, Thomas G. Allison, Christopher M. Reid, James N. Kirkpatrick

**Affiliations:** ^1^Vietnam National Heart Institute, Bach Mai Hospital, Hanoi, Vietnam; ^2^Department of Internal Medicine, VNU-University of Medicine and Pharmacy, Hanoi, Vietnam; ^3^Department of Cardiology, Hanoi Medical University, Hanoi, Vietnam; ^4^Department of Cardiovascular Medicine, Mayo Clinic, Rochester, MN, United States; ^5^School of Population Health, Curtin University, Perth, WA, Australia; ^6^School of Public Health and Preventive Medicine, Monash University, Melbourne, VIC, Australia; ^7^Cardiovascular Division, Department of Medicine, University of Washington Medical Center, Seattle, WA, United States; ^8^Department of Bioethics and Humanities, University of Washington Medical Center, Seattle, WA, United States

**Keywords:** chronic heart failure, body mass index, obesity paradox, underweight, mortality, hospitalization

## Abstract

**Background:**

Insufficient data exists regarding the relationship between body mass index (BMI) and the prognosis of chronic heart failure (CHF) specifically within low- and middle-income Asian countries. The objective of this study was to evaluate the impact of BMI on adverse outcomes of ambulatory patients with CHF in Vietnam.

**Methods:**

Between 2018 and 2020, we prospectively enrolled consecutive outpatients with clinically stable CHF in an observational cohort, single-center study. The participants were stratified according to Asian-specific BMI thresholds. The relationships between BMI and adverse outcomes (all-cause death and all-cause hospitalization) were analyzed by Kaplan–Meier survival curves and Cox proportional-hazards model.

**Results:**

Among 320 participants (age 63.5 ± 13.3 years, 57.9% male), the median BMI was 21.4 kg/m^2^ (IQR 19.5–23.6), and 10.9% were underweight (BMI <18.50 kg/m^2^). Over a median follow-up time of 32 months, the cumulative incidence of all-cause mortality and hospitalization were 5.6% and 19.1%, respectively. After multivariable adjustment, underweight patients had a significantly higher risk of all-cause mortality than patients with normal BMI (adjusted hazard ratios = 3.03 [95% CI: 1.07–8.55]). Lower BMI remained significantly associated with a worse prognosis when analyzed as a continuous variable (adjusted hazard ratios = 1.27 [95% CI: 1.03–1.55] per 1 kg/m^2^ decrease for all-cause mortality). However, BMI was not found to be significantly associated with the risk of all-cause hospitalization (*p* > 0.05).

**Conclusion:**

In ambulatory patients with CHF in Vietnam, lower BMI, especially underweight status (BMI < 18.5 kg/m^2^), was associated with a higher risk of all-cause mortality. These findings suggest that BMI should be considered for use in risk classification, and underweight patients should be managed by a team consisting of cardiologists, nutritionists, and geriatricians.

## Introduction

Heart failure (HF) stands as a prominent contributor to global morbidity and mortality and has become a rapidly growing healthcare burden in Vietnam (1–3). Overweight and obesity, characterized by a higher body mass index (BMI), have been identified as important risk factors for the development of HF due to negative changes in hemodynamics and cardiac structure (4). The number of overweight/obese people is expected to rise rapidly, especially in low- and middle-income countries (5). The improved living standards in the Asia-Pacific region have led to lifestyle changes, including adopting unhealthy diets and physical inactivity (6, 7). This contributes to a dramatic rise in the prevalence of overweight/obesity in the region. Although Vietnam has the lowest obesity rate in Southeast Asia, it has experienced the fastest increase in obesity prevalence (7, 8). A study conducted in Hanoi by Wall et al. revealed that both underweight and overweight were prevalent among urban adults (9).

Some previous longitudinal studies have shown that obese patients with chronic heart failure (CHF) have a lower risk of mortality, which is termed the “obesity paradox” (10–12). In addition, these findings also suggest that individuals who are underweight may have a higher risk of mortality compared to those who are normal or overweight (12, 13). Nevertheless, a recent study published in 2023 found no supporting evidence for the protective effect of obesity, raising questions about the existence of the “obesity paradox” (14). On the other hand, the prognosis of CHF is also influenced by the management of post-discharged patients, which may confound the relationship between BMI and clinical outcomes. Multiple large clinical trials have confirmed that the implementation of guideline-directed medication therapy (GDMT) effectively decreases mortality rates, reduces heart failure (HF) hospitalizations, and enhances functional capacity (15). The association between BMI and the outcome of CHF may become more apparent when there are fewer confounding factors present in patients who are stable and managed within a heart failure program. Currently, there are limited available data on the relationship between BMI and the outcomes of HF patients in low- and middle-income Asian countries, particularly in Vietnam. To address this gap, we conducted a prospective cohort study to investigate the association between BMI and the prognosis of ambulatory CHF patients at the Vietnam National Heart Institute (VNHI), Bach Mai Hospital, Hanoi, Vietnam.

## Methods

### Study population

Our prospective study enrolled consecutive patients with stable CHF in the specialized HF program at Outpatient Department (ODP), VNHI, Bach Mai Hospital from April 2018 to September 2020. Inclusion criteria were as follows: (1) age ≥ 18 years, (2) diagnosis of CHF was established according to the European Society of Cardiology (ESC) guidelines (1, 16), (3) comprehensive clinical and laboratory tests were conducted at the ODP, VNHI during the first appointment, (4) patients were treated with GDMT for HF with optimal doses of disease-modifying drugs, and (5) patients were advised on diet and lifestyle modification during the treatment duration. We excluded patients with acute HF, acute exacerbation of CHF, and other severe medical conditions. Patients who did not comply with the GDMT in the HF program were also excluded. In cases where the patient or patient’s family members could not be contacted, we excluded patients due to loss to follow-up.

### Data collection and definitions

#### Baseline survey

The cardiologists of VNHI’s HF program recorded patients’ demographics, medical history, clinical characteristics, medications, and HF risk factors on a pre-established data collection form (DCF). Systolic/diastolic blood pressure was measured with an automatic sphygmomanometer by trained nurses. Data on electrocardiograms, echocardiography, and laboratory tests were also collected upon presentation. The estimated glomerular filtration rate (eGFR) was determined by employing the Modification of Diet in Renal Disease (MDRD) formula for calculation (17). Anemia was defined based on the criteria established by the World Health Organization (hemoglobin levels <13.0 g/dL for males and < 12.0 g/dL for females) (18). Patients were classified as current smokers if they were currently smoking or had smoked for at least 5 years and quit smoking within 1 year before the study. Alcohol consumption was defined as a weekly alcohol intake of ≥7 standard drinks (19). The HF subtypes were determined based on the left ventricle ejection fraction (LVEF): HF with reduced LVEF (HFrEF, LVEF ≤40%), HF with mildly reduced LVEF (HFmrEF, 41% ≤ LVEF ≤49%), and HF with preserved LVEF (HFpEF, LVEF ≥50%) (1).

BMI was calculated as body weight (kg) divided by squared height (m^2^) (20). Weight was measured by a vertical scale R62-120 and was recorded to the nearest 0.1 kg. Height was measured using a metric stadiometer attached to a wall and calibrated according to local policy, with measurements taken to the nearest 1 cm. The process of measuring weight and height was carried out by the HF nurses. Participants were classified into four groups based on Asian-specific BMI cutoff points using WHO classifications: underweight (BMI <18.5 kg/m^2^), normal (BMI 18.5–22.9 kg/m^2^), overweight (BMI 23.0–24.9 kg/m^2^) and obesity (BMI ≥25.00 kg/m^2^) (21, 22).

#### Outcomes and follow-up

A three-year follow-up survey was designed to collect data on outcomes of interest, including all-cause mortality and all-cause hospitalizations. All-cause mortality included all deaths which were determined by hospital death records or confirmed by patients’ family members. All-cause hospitalization was defined as hospital admission for any cause. Our patients were followed by dedicated cardiologists and nurses via direct contact in the outpatient setting if the participants attended in-person visits at VNHI. Alternatively, phone interviews were conducted when the participants were unavailable for an in-person visit. The information was collected by trained nurses using pre-written questionnaires in the DCFs.

### Statistical analysis

Continuous variables were presented as either the mean ± standard deviation (SD) or the median (interquartile range, IQR), while categorical variables were expressed as the number of subjects (%) in the dataset. Between-group differences were evaluated using Student’s t-test, Mann–Whitney U test, ANOVA test, or Kruskal Wallis test for continuous variables as appropriate. The Chi-square (χ2) test or Fisher’s exact test was used to compare categorical variables. The unadjusted risks of the clinical outcomes (all-cause mortality and hospitalization) across the BMI groups were assessed using the Kaplan–Meier survival curve analysis with the intergroup differences assessed by the log-rank test. Given the low occurrence of events among individuals classified as overweight or obese, we categorized the body mass index (BMI) into three groups: underweight (BMI <18.5 kg/m^2^), normal range (BMI 18.5–22.9 kg/m^2^), and overweight/obesity (BMI ≥23.0 kg/m^2^) to evaluate the impact of BMI on the outcomes of interest. Univariable and multivariable Cox proportional hazard regression analyses were then performed to evaluate the hazard ratios (HR), adjusted HRs, and their corresponding 95% confidence intervals (95% CI) using BMI as either a continuous or categorical variable. To avoid overfitting, only variables which reached a *p*-value less than 0.10 (in univariable analysis) were included in the multivariable analysis. In addition, sensitivity analysis was performed to assess whether using LVEF or heart failure classification in the multivariate Cox regression model caused significant change in the result. All tests were two-sided, and a value of *p* < 0.05 was considered statistically significant. All analyses were performed using Stata MP version 17.0 (Stata, College Station, TX, United States).

#### Ethics approval

Ethical approval for the study was obtained from the ethics committee of Bach Mai Hospital. Each patient was given an information sheet that explicitly outlined the study’s objectives, procedures, and participant rights. Written informed consent was obtained from all participants. All patient-identifying information was coded and maintained in strict confidentiality.

## Results

### Baseline patients’ characteristics

**Figure 1:**
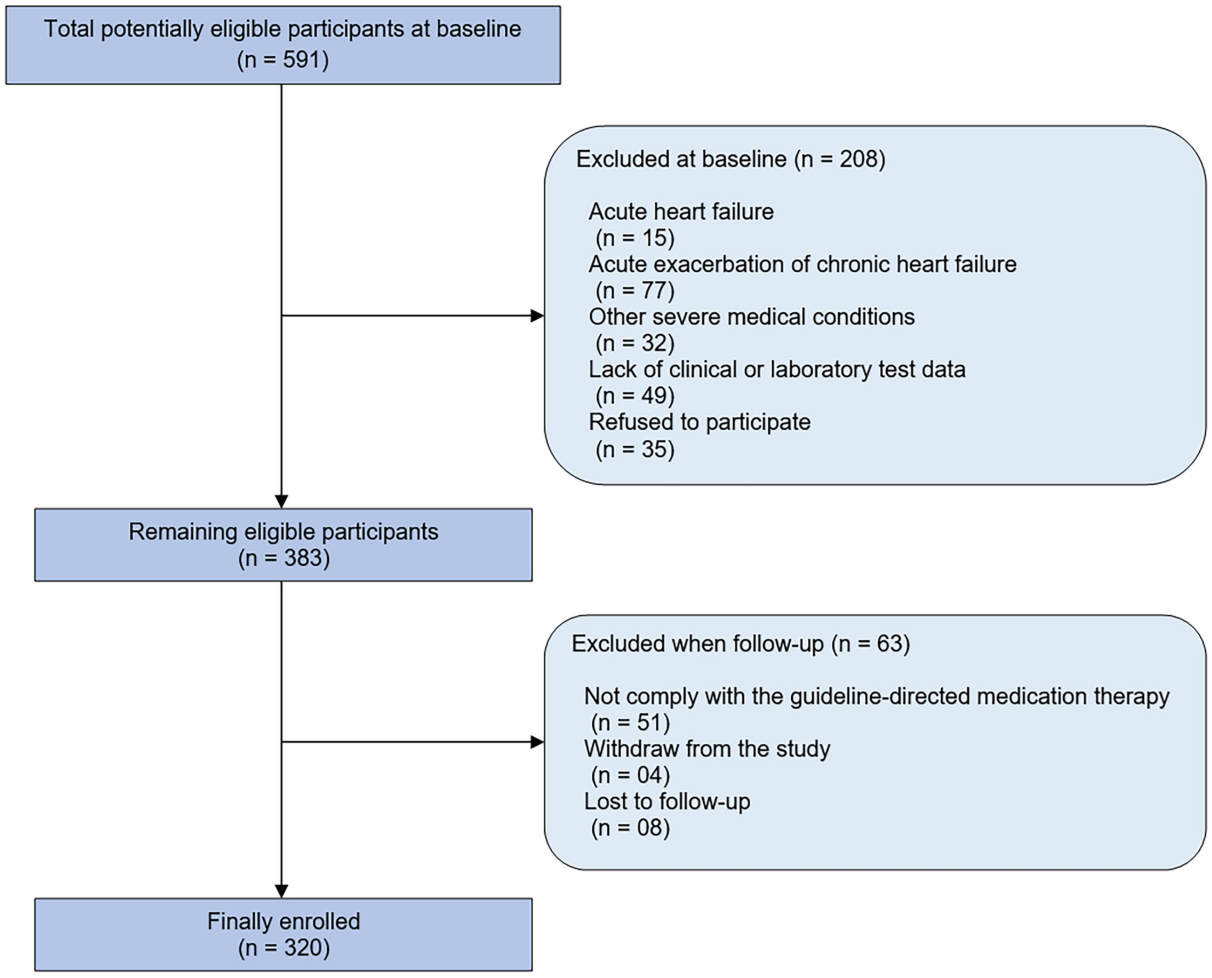
The study population flow diagram.

**Figure 2:**
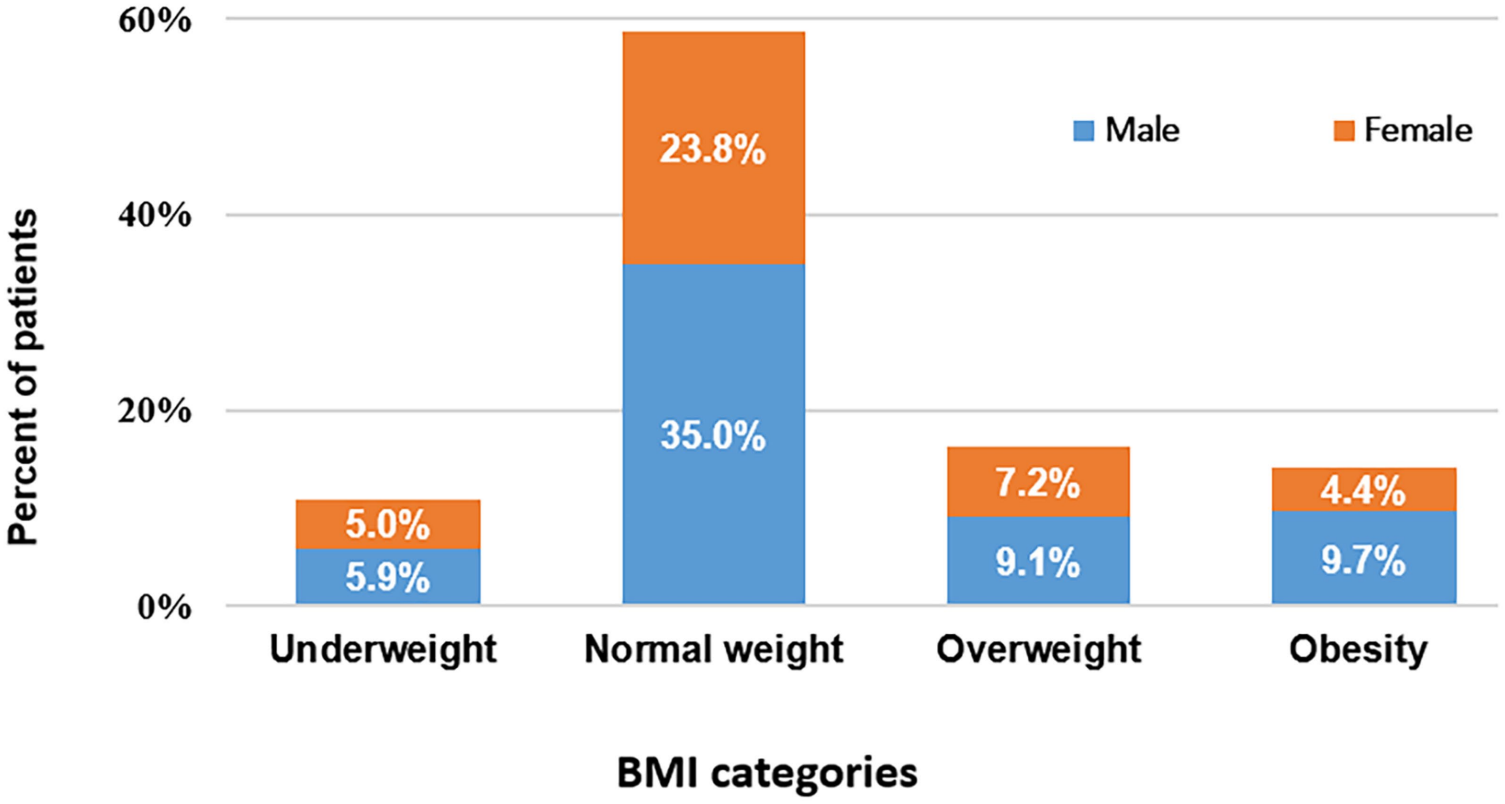
Distribution of genders by BMI categories.

From April 2018 to September 2020, a total of 320 outpatients with CHF were included in our study, 124 patients were excluded due to their baseline medical condition, and eight patients were excluded because of loss to follow-up. The detailed population flow diagram may be found in Figure 1. The overall mean age was 63.5 ± 13.3 years, and BMI (median, IQR) was 21.4 (19.5–23.6) kg/m^2^. Figure 2 shows the sex distribution across the BMI categories. There were 188 patients with BMI within the normal range, which accounted for 58.8% of patients: 112 men (35.0%), 76 women (23.8%); 35 underweight patients (10.9%): 19 men (5.9%), six women (5.0%); 52 overweight patients (16.3%): 29 men (9.1%), 23 women (7.2%); and 45 obese patients (14.1%): 31 men (9.7%), 14 women (4.4%). Notably, a majority of obese patients (42; 13.1%) had obesity class I (25.0 ≤ BMI ≤29.9), and there were only three patients (0.9%) had obesity class II (BMI ≥30.0 kg/m^2^) (21). Among them, there were no patients with a BMI exceeding 31.0 kg/m^2^. The baseline characteristics of the entire study population and by categories of BMI are shown in Table 1. There were no significant differences across the BMI groups regarding socio-demographics, dyslipidemia, diabetes, hypertension, smoking, drinking, HF duration, blood pressure, heart rate, NYHA functional class, NT-proBNP, renal function, the prevalence of atrial fibrillation, and the use of disease-modifying medications (*p* > 0.05 for all). Anemia was more common in the underweight group than in the higher BMI groups, but the differences were not statistically significant (*p* > 0.05).

**Table 1:** Baseline characteristics of study participants stratified by BMI categories.

Characteristics		Total (*n* = 320)	Body mass index				*p*-value
			Underweight (<18.5 kg/m^2^) (*n* = 35)	Normal range (18.5–22.9 kg/m^2^) (*n* = 188)	Overweight (23.0–24.9 kg/m^2^) (*n* = 52)	Obese (≥25.0 kg/m^2^) (*n* = 45)	
		$\bar{X}$ ± SD / median (IQR)/*n* (%)					
Demographics and clinical characteristics							
Age (years)		63.5 ± 13.3	64.9 ± 18.0	63.7 ± 13.6	63.0 ± 9.7	62.2 ± 11.4	0.811
Gender	Male	191 (59.7)	19 (54.3)	112 (59.6)	29 (55.8)	31 (68.9)	0.512
	Female	129 (40.3)	16 (45.7)	76 (40.4)	23 (44.2)	14 (31.1)	
NYHA functional class	I	157 (49.1)	15 (42.9)	97 (51.6)	28 (53.8)	17 (37.8)	0.290
	II	117 (36.5)	13 (37.1)	67 (35.6)	19 (36.5)	18 (40.0)	0.960
	III	46 (14.4)	7 (20.0)	24 (12.8)	5 (9.6)	10 (22.2)	0.208
SBP (mmHg)		120.9 ± 11.6	119.6 ± 10.2	120.6 ± 12.2	122.1 ± 11.4	121.8 ± 10.7	0.499
DBP (mmHg)		76.2 ± 8.5	74.9 ± 8.2	76.3 ± 8.8	76.3 ± 8.4	76.6 ± 7.7	0.907
Heart rate (b.p.m)		74.3 ± 6.8	75.1 ± 6.1	73.9 ± 7.1	74.5 ± 6.0	74.9 ± 7.1	0.678
HF phenotype							
HFrEF (LVEF<40%)		103 (32.2)	16 (45.7)	62 (33.0)	14 (26.9)	11 (24.4)	0.183
HFmrEF (LVEF 41–49%)		75 (23.4)	8 (22.9)	32 (17.0)	18 (34.6)	17 (38.7)	0.004*
HFpEF (LVEF≥50%)		142 (44.4)	11 (31.4)	94 (50.0)	20 (38.5)	17 (37.0)	0.096
Medical history							
Hypertension		130 (40.6)	11 (31.4)	76 (40.4)	25 (48.1)	18 (40.0)	0.493
Diabetes		64 (20.0)	5 (14.3)	35 (18.6)	12 (23.1)	12 (26.7)	0.476
Dyslipidemia		125 (39.1)	12 (34.3)	73 (38.8)	22 (42.3)	18 (40.0)	0.900
Anemia		19 (5.9)	4 (11.4)	10 (5.3)	4 (7.7)	1 (2.2)	0.315
Atrial fibrillation		25 (7.8)	2 (5.7)	15 (8.0)	2 (3.8)	6 (13.3)	0.396
HF duration	≤5 years	290 (90.6)	32 (91.4)	165 (87.8)	49 (94.2)	44 (97.8)	0.159
	>5 years	30 (9.4)	3 (8.6)	23 (12.2)	3 (5.8)	1 (2.2)	
Current smoker		233 (72.8)	27 (77.1)	136 (72.3)	39 (75.0)	31 (68.9)	0.843
Alcohol consumption		242 (75.6)	26 (74.3)	142 (75.5)	43 (82.7)	31 (68.9)	0.466
Laboratory tests							
NT-ProBNP (pg/mL)		82.1 (59.2–105.8)	88.0 (60.8–117.8)	80.0 (59.8–104.5)	83.9 (54.0–101.6)	80.3 (59.1–105.8)	0.609
LDL cholesterol (mmol/L)		2.2 (1.6–2.8)	2.2 (1.7–2.7)	2.2 (1.5–2.8)	2.3 (1.6–3.0)	2.3 (2.1–3.1)	0.139
Ure (mmol/L)		5.9 (4.8–7.5)	6.7 (4.8–7.9)	6.1 (4.9–7.5)	5.4 (4.5–6.4)	5.9 (5.2–7.3)	0.115
Creatinine (μmol/L)		82.0 (69.0–97.0)	76.0 (66.0–95.0)	82.0 (70.0–97.0)	79.0 (67.5–91.5)	86.0 (68.0–105.0)	0.324
eGFR (mL/min/1.73 m^2^)		79.0 ± 23.3	80.0 ± 27.6	77.7 ± 22.1	82.7 ± 24.1	79.0 ± 23.7	0.586
Acid uric (μmol/L)		397.1 ± 115.4	406.2 ± 146.6	383.5 ± 108.1	400.3 ± 119.6	433.8 ± 103.4	0.152
Echocardiographic parameters							
LVEF (%)		47.9 ± 14.1	43.7 ± 13.9	48.4 ± 14.1	49.1 ± 15.2	48.1 ± 12.7	0.286
LVEDD (mm)		53.0 ± 9.1	52.3 ± 10.1	52.5 ± 9.2	53.4 ± 9.2	54.9 ± 8.0	0.416
RVEDD (mm)		22.1 ± 3.7	21.8 ± 3.7	22.3 ± 3.8	21.4 ± 3.4	22.4 ± 3.3	0.577
Medication							
Use of ACEi/ARB		176 (55.0)	21 (60.0)	105 (55.9)	29 (55.8)	21 (46.7)	0.650
Use of ARNI		104 (32.6)	14 (40.0)	61 (32.4)	17 (32.7)	12 (27.3)	0.706
Use of β-Blockers		159 (49.7)	12 (34.3)	92 (48.9)	29 (55.8)	26 (57.8)	0.152
Use of MRAs		82 (25.6)	11 (31.4)	49 (26.1)	13 (25.0)	9 (20.0)	0.713
Use of SGLT2i		50 (15.6)	3 (8.6)	27 (14.4)	9 (17.3)	11 (24.4)	0.230

SD, standard deviation; IQR, interquartile range; NYHA, New York Heart Association; SBP, systolic blood pressure; DBP, diastolic blood pressure; HF, heart failure; LVEF, left ventricular ejection fraction; HFrEF, HF with reduced LVEF; HFmrEF, HF with mildly reduced LVEF; HFpEF, HF with preserved LVEF; eGFR, estimated glomerular filtration rate; LVEDD, Left ventricular end-diastolic diameter; RVEDD, right ventricular end-diastolic diameter; ACEi/ARB, angiotensin-converting enzyme inhibitors/angiotensin receptor blockers; ARNI, angiotensin receptor-neprilysin inhibitor; MRAs, mineralocorticoid receptor antagonists; SGLT2i, sodium glucose cotransporter 2 inhibitor. *p < 0.05.

There was no significant difference observed in the prevalence of HFrEF and HFpEF among the various BMI categories (*p* > 0.05). Nevertheless, HFmrEF prevalence was more frequent in the overweight and obesity groups (34.6 and 39.1%, respectively) than in the underweight and the normal range groups (22.9% and 17.0%, respectively). Additionally, the HFmrEF tended to have a higher mean BMI (22.5 ± 3.2) compared to the HFrEF and HFpEF groups (21.4 ± 3.2 and 21.5 ± 2.5, respectively) with a *p*-value of less than 0.05. Echocardiographic left ventricular as well as right ventricular end-diastolic diameters were not different between groups (*p* > 0.05 for both).

### Impact of body mass index on the outcomes

**Figure 3:**
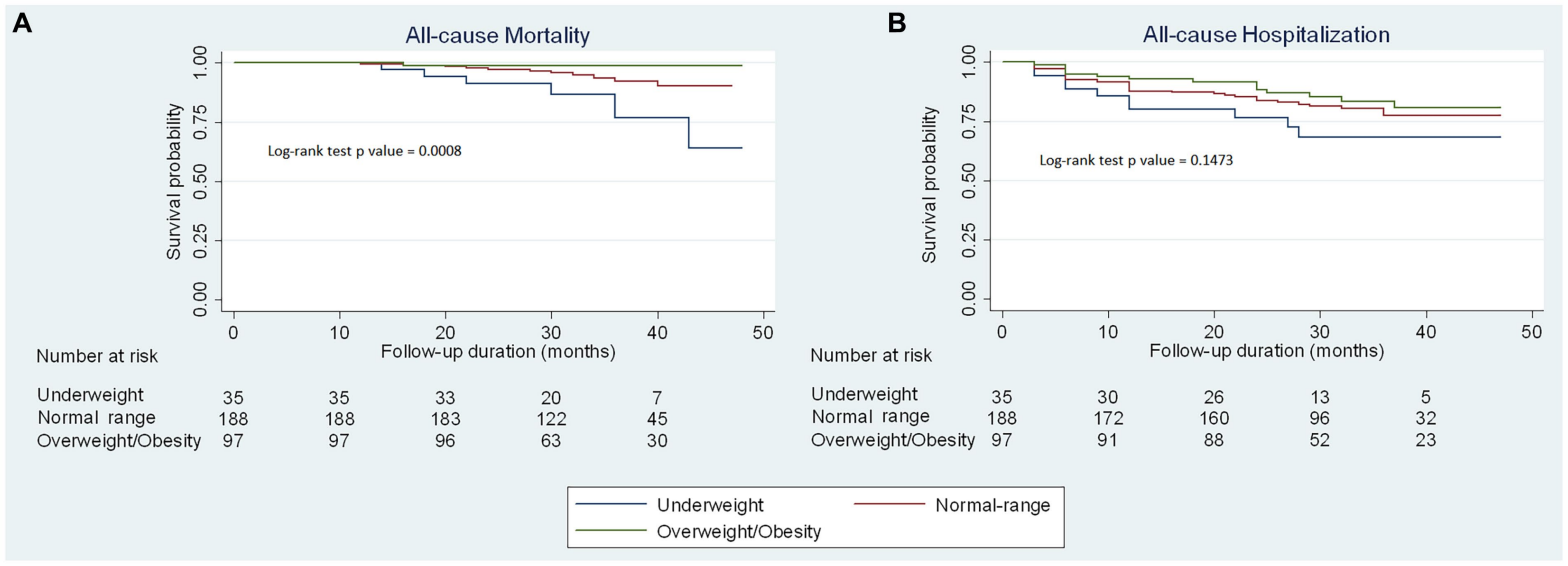
Kaplan–Meier curves of clinical outcomes stratified by BMI categories. Kaplan–Meier curves to predict **(A)** all-cause mortality and **(B)** all-cause hospitalization for outpatients with chronic heart failure by body mass index categories.

During a median follow-up time of 32 months (IQR 27–40 months), there were 18 patients (5.6%) with all-cause death and 61 patients (19.1%) with all-cause hospitalization. The incident mortality rate was 2.1% per 100 person-years in the overall study population, 6.6% among underweight patients, 2.1% among normal range patients, and 0.4% among overweight/obesity patients. Kaplan–Meier curves illustrating the cumulative probability of time to occurrence of all-cause mortality and all-cause hospitalization are represented in Figure 3. The underweight group showed a markedly higher incidence of all-cause mortality compared to both the normal range and overweight groups, with statistical significance (*p* = 0.0008). However, there was no significant difference between the three groups regarding all-cause hospitalization (*p* = 0.1473).

Table 2 displays the results of univariable Cox regression analyses of predictive factors for all-cause mortality and all-cause hospitalization. Older age, worse NYHA functional class, lower LVEF, eGFR <60 (mL/min/1.73 m^2^), and lower BMI (both as a continuous variable and as a categorial variable) were significantly associated with higher mortality risk while only age and sex were found to be significantly associated with the risk of all-cause hospitalization. Nevertheless, multivariable Cox regression analyses (Table 3) indicated that only age (adjusted HR = 1.08 [95% CI: 1.03–1.13] in model 1 and adjusted HR = 1.08 [95% CI: 1.04–1.13] in model 2) and BMI were independent predictors of all-cause mortality. Specifically, when used as a continuous variable, model 1 demonstrated an inverse relationship between BMI and the risk of all-cause mortality (adjusted HR per 1 kg/m^2^ decrease: 1.27 [95%CI: 1.03–1.55, *p* = 0.023]). When used as a categorial variable, the underweight group (BMI <18.5 kg/m^2^) was significantly associated with a higher mortality risk (adjusted HR = 3.03 [95% CI: 1.07–8.55, *p* = 0.036]) compared to the normal range group (BMI 18.5–22.9 kg/m^2^). However, the risk for all-cause mortality was not significantly different between the overweight/obese group (BMI ≥23.0 kg/m^2^) and the normal range group, although there was a trend towards a lower risk of death in overweight/obese compared to normal weight patients (adjusted HR = 0.22 [95% CI: 0.03–1.79, *p* = 0.159]). Similar results were obtained in a sensitivity analysis where the HF phenotype variable was utilized in place of the LVEF variable (Supplementary Table S1).

**Table 2:** Univariable cox regression analysis of predictors for clinical outcomes.

Variables		All-cause mortality		All-cause hospitalization	
		HR (95% CI)	*p*-value	HR (95% CI)	*p*-value
Age (increase 1 year)		1.11 (1.06–1.15)	<0.001*	1.03 (1.01–1.05)	0.004*
Sex (female)		1.25 (0.49–3.17)	0.646	1.75 (1.06–2.90)	0.029*
BMI (increase 1 kg/m^2^)		1.34 (1.11–1.63)	0.003*	1.07 (0.98–1.18)	0.117
BMI categories	Normal range (BMI 18.5–22.9 kg/m^2^)	1.0 (References)	–	1.0 (References)	–
	Underweight (BMI <18.5 kg/m^2^)	3.26 (1.20–8.83)	0.020*	1.67 (0.83–3.37)	0.150
	Overweight/Obesity (BMI ≥23.0 kg/m^2^)	0.17 (0.02–1.29)	0.085	0.77 (0.42–1.41)	0.397
NYHA funtional class	I	1.0 (References)	–	1.0 (References)	–
	II	1.59 (0.48–5.20)	0.447	1.37 (0.80–2.36)	0.258
	III	4.50 (1.42–14.19)	0.010*	1.15 (0.537–2.45)	0.723
LVEF (increase 1%)		0.95 (0.92–0.99)	0.008*,^a^	1.01 (0.99–1.03)	0.458
HF subtype	HFrEF (LVEF<40%)	1.0 (References)	–	1.0 (References)	–
	HFmrEF (LVEF 41–49%)	0.39 (0.11–1.41)	0.150	1.06 (0.53–2.14)	0.864
	HFpEF (LVEF≥50%)	0.37 (0.13–1.07)	0.067^a^	1.20 (0.66–2.15)	0.551
Hypertension		1.44 (0.57–3.62)	0.443	1.21 (0.73–2.00)	0.468
Diabetes mellitus		0.75 (0.22–2.58)	0.646	1.43 (0.81–2.53)	0.217
Anemia		2.09 (0.48–9.11)	0.326	0.80 (0.25–2.55)	0.704
NT-proBNP (increase 10 pg./mL)		1.04 (0.95–1.15)	0.408	1.03 (0.98–1.09)	0.253
eGFR (<60 mL/min/1,73m^2^)		3.56 (1.41–8.98)	0.007*	1.24 (0.70–2.23)	0.461
Use of beta-blockers		1.05 (0.42–2.64)	0.922	0.80 (0.48–1.33)	0.389
Use of RAS blockers		1.96 (0.45–8.52)	0.371	0.83 (0.45–1.53)	0.543
Use of MRA		1.15 (0.41–3.23)	0.791	1.52 (0.89–2.60)	0.122
Use of SGLT-2i		1.42 (0.47–4.31)	0.540	1.03 (0.52–2.02)	0.940
Current smoker		0.75 (0.28–1.99)	0.556	1.19 (0.66–2.13)	0.565
Alcohol consumption		0.88 (0.31–2.48)	0.810	1.39 (0.74–2.61)	0.310

HR, hazard ratio; 95% CI, 95% confidence interval; BMI, body mass index; NYHA, New York Heart Association; LVEF, left ventricular ejection fraction; HF, heart failure; HFrEF, HF with reduced LVEF; HFmrEF, HF with mildly reduced LVEF; HFpEF, HF with preserved LVEF; eGFR, estimated glomerular filtration rate; RAS, renin-angiotensin-system; MRAs, mineralocorticoid receptor antagonists; SGLT2i, sodium glucose cotransporter 2 inhibitor. *p < 0.05; ^a^HF subtype (with value of *p* < 0.10) was excluded from the multivariable analysis due to the concern of collinearity with LVEF (%).

**Table 3:** Multivariable cox regression analysis of predictors for clinical outcomes.

Variables		Multivariable model #1			Multivariable model #2		
		Adjusted HR	95% CI	*p*-value	Adjusted HR	95% CI	*p*-value
All-cause mortality							
Age (increase 1 year)		1.08	1.03–1.13	<0.001*	1.08	1.04–1.13	<0.001*
NYHA funtional class	I	1.0 (Reference)			1.0 (Reference)		
	II	1.33	0.39–4.59	0.650	1.44	0.42–4.91	0.559
	III	2.48	0.52–11.83	0.255	2.22	0.47–10.48	0.314
LVEF (decrease 1%)		1.01	0.96–1.06	0.608	1.01	0.97–1.06	0.564
eGFR (<60 mL/min/1,73m^2^)		1.49	0.53–4.19	0.452	1.57	0.55–4.44	0.396
BMI (decrease 1 kg/m^2^)		1.27	1.03–1.55	0.023*	–	–	–
BMI categories	Normal range (BMI 18.5–22.9 kg/m^2^)	–	–	–	1.0 (Reference)		
	Underweight (BMI <18.5 kg/m^2^)	–	–	–	3.03	1.07–8.55	0.036*
	Overweight/Obesity (BMI ≥23.0 kg/m^2^)	–	–	–	0.22	0.03–1.79	0.159
All-cause hospitalization							
Age (increase 1 year)		1.03	1.01–1.05	0.007*	1.03	1.01–1.05	0.007*
Sex (female)		1.68	1.01–2.78	0.044*	1.68	1.02–2.78	0.043*
BMI (increase 1 kg/m^2^)		0.94	0.86–1.03	0.215	–	–	–
BMI categories	Underweight (BMI <18.5 kg/m^2^)	–	–	–	1.60	0.79–3.23	0.193
	Normal range (BMI 18.5–22.9 kg/m^2^)	–	–	–	1.0 (Reference)		
	Overweight/Obesity (BMI ≥23.0 kg/m^2^)	–	–	–	0.81	0.45–1.49	0.504

Multivariable model #1: BMI as a continuous variable. Multivariable model #2: BMI as a categorial variable. HR, Hazard ratio; 95% CI, 95% Confidence interval; NYHA, New York Heart Association; LVEF, Left ventricular ejection fraction; eGFR, estimated Glomerular filtration rate; BMI, Body mass index. *p < 0.05.

Notably, BMI was not found to be significantly associated with the risk of all-cause hospitalization (adjusted HR per 1 kg/m^2^ decrease: 1.06 [95%CI: 0.97–1.16, *p* = 0.215] in model 1 and adjusted HR = 1.60 [95% CI: 0.79–3.23, *p* = 0.193] for underweight group/adjusted HR = 0.81 [95% CI: 0.45–1.49, *p* = 0.504] for overweight/obesity group in model 2) in multivariable analyses. Nevertheless, older age (adjusted HR per 1 year increase: 1.03 [95%CI: 1.01–1.05] in both models) and female sex (adjusted HR = 1.68 [95% CI: 1.01–2.78] in model 1 and adjusted HR = 1.68 [95% CI: 1.02–2.78] in model 2) remained significant predictors of all-cause hospitalization.

## Discussion

To our knowledge, this is the first prospective study investigating the association between BMI and clinical outcomes among HF outpatients in Vietnam. The major findings of the present study were as follows: (1) A lower body mass index, particularly being underweight, demonstrated a significant association with a higher incidence of all-cause mortality; (2) There was a trend towards a lower risk of death in overweight/obese compared to normal weight patients, but it is not significant; and (3) BMI did not show a significant association with the risk of all-cause hospitalization.

### BMI and all-cause mortality risk

The phenomenon known as the obesity paradox in patients with heart failure (HF) refers to the noteworthy observation of a lower risk of mortality and other adverse events among individuals with higher body mass index (BMI), in contrast to those with lower BMI who exhibit a worse prognosis (23, 24). This phenomenon is counterintuitive to the well-known associations between obesity and recognized risk factors for cardiovascular disease, including diabetes, high blood pressure, high low-density lipoprotein (LDL) cholesterol, and heightened chronic inflammation, but is observed in several chronic conditions, particularly those with heart failure (25).

Horwich et al. (23) were the pioneering authors who reported on this paradox in a prospective, single-center cohort study involving 1,203 patients diagnosed with systolic heart failure. The results revealed that higher BMI was not associated with increased mortality but may be a protective factor for improved survival (23). In this study, the underweight HF patients exhibited the worst prognosis, with the normal-range BMI group following suit. After that time, numerous studies have reported similar findings. For example, the largest retrospective analysis investigating the association between body mass index (BMI) and in-hospital mortality involved over 108,000 patients diagnosed with HFrEF and HFpEF from the ADHERE study. This study demonstrated a significant association, as a 10% increase in mortality was observed with each five-unit decrease in BMI (26). A more recent meta-analysis of published research on BMI and HF outcomes included 22,807 hospitalized patients with both HFrEF and HFpEF and showed that those at the highest risk of the three outcomes had low BMI (<20 kg/m^2^) while those at lowest risk were in the overweight category (BMI 25–29.9 kg/m^2^), after a median follow-up of 2.85 years (13). Furthermore, a negative correlation between mortality and BMI was also observed in Asian heart failure populations. An analysis of a Korean HF registry that included 4,146 patients hospitalized for acute HF showed that a lower BMI is associated with an increased risk of mortality, and this relationship is observed consistently across various comorbidities and heart failure subtypes (27). The data from the PURSUIT-HFpEF registry in Japan, which included 846 patients, also determined that being underweight was significantly associated with poor prognosis in HFpEF (28). In addition, a collaborative meta-analysis, which included 8 studies involving 7,224 East Asian HF patients, also demonstrated a strong inverse association between BMI and mortality (29).

Despite abundant evidence supporting an inverse association between BMI and mortality in HF, most of the studies were conducted in Western and high-income Asian countries. Therefore, acquiring additional data on the relationship between BMI and the outcomes of HF patients in low- and middle-income Asian countries, characterized by lower mean body mass indices, a higher prevalence of underweight patients, and differing eating habits and nutritional statuses compared to those in Western countries, could make a valuable contribution to the existing literature (22, 28). In this study, after adjusting for appropriate confounders, the results showed that lower BMI (aHR per 1 kg/m^2^ decrease: 1.27 [95% CI: 1.03–1.55]), especially underweight status (aHR = 3.03 [95% CI: 1.07–8.55]), was independently associated with a higher risk of all-cause mortality. However overweight/obesity status was not significantly linked to the outcome (aHR = 0.22 [95% CI: 0.03–1.79]).

A recent study, based on PARADIGM-HF data, yielded results similar to ours, as it found no survival benefits associated with obesity while the alterative anthropometric indices (e.g., waist-to-height ratio) was significantly associated with the primary outcome (14). This result can be explained by the close impact of waist-to-height ratio (WHtR) on cardiorespiratory fitness (CRF), which is an important prognostic factor for HF (30). Specifically, in patients with abdominal obesity and a high WHtR, the increase in intra-abdominal pressure due to chest expansion and diaphragm descent during inspiration may contribute to a decrease in CRF (30). Furthermore, a recent study has demonstrated that WHtR and waist circumference (WC) are more strongly associated with VO_2_ max than BMI (31). Notably, it has been also suggested that the obesity paradox may be more pronounced in HF patients with low CRF (32).

The blunting of the obesity paradox in this study may also be attributed to several other factors. First, the study population did not show substantial differences in baseline characteristics across BMI subgroups, specifically in relation to potential confounding variables such as age, gender, comorbidities, smoking status, and optimal medications for the treatment of heart failure. This condition is important in minimizing the risk of bias when evaluating the influence of BMI since overweight and obese individuals are more likely to have obesity-related symptoms, including dyspnea, fatigue, and leg swelling, earlier and more frequently, and thus are monitored more closely by physicians (33). VNHI’s outpatient heart failure program comprehensively evaluates and monitors all patients, enabling physicians to remain current on their patient’s conditions and make timely treatment adjustments. It ensures adherence to guideline-directed medical therapy in the majority of patients. Additionally, several studies demonstrated that patients who were managed in specialized HF centers were more likely to receive GDMT and target doses of disease-modifying drugs and, as a result, likely had better prognoses compared to those in general cardiology outpatient clinics (34). The survival and readmission rates were significantly improved, especially in patients with home telemonitoring by trained medical staff, which may also make it difficult to evaluate the differences in mortality among BMI groups (35, 36). Second, the patients with obesity class I and II in this study made up significantly lower proportions (13.1% and 0.9%) of the total population, compared to previous studies, and therefore the sample size may be insufficiently powered to detect the survival benefits of high BMI on mortality. Third, differences in race and other clinical characteristics of the enrolled patients between our study and previous studies may impact the effects of BMI. Finally, the 32-month median follow-up period in this study may not provide adequate time to assess mortality events, especially if there are time-dependent changes in the interaction between BMI and HF and their effects on total mortality (37).

Several hypotheses explaining the mechanistic links between underweight status and poor outcomes have been proposed. In heart failure, it has been demonstrated that being underweight is characterized by a decrease in muscle mass and function (sarcopenia), as well as a reduction in tissue mass (cachexia) (38, 39). Sarcopenia can be attributed to various factors, including the natural process of aging, lack of physical activity leading to disuse muscle atrophy, malnutrition, and disease (39). Cardiac cachexia arises from the imbalance between protein synthesis and protein degradation, and it can also stem from impaired nutrient absorption (40). Data from Studies Investigating Comorbidities Aggravating Heart Failure (SICA-HF) revealed that sarcopenia and cachexia are prevalent in patients with CHF (41–43). Both of these conditions result in unintentional weight loss, which has been demonstrated to be associated with an increased risk of mortality in chronic heart failure (44). Moreover, another study involving 268 ambulatory HF patients from SICA-HF with a mean follow-up of 67.2 ± 28.02 months revealed that muscle wasting is a strong predictor of death (45). Furthermore, the prevalence of acute cardiovascular events in underweight patients with CHF may be higher than in those with normal weight because of immunodeficiency, which leads to susceptibility to severe infection and frailty (46). In addition to the mentioned mechanisms, the lack of protective effects of obesity may also contribute to poor outcomes in underweight patients. The increase in lean mass among obese patients may significantly contribute to improved long-term outcomes by enabling greater cardio-respiratory fitness. Additionally, surplus adiposity appears to induce the production of pro-inflammatory cytokines, potentially offering greater protective effects in overweight and obese patients with lower levels of systemic inflammation. Moreover, excess adipose tissue mobilizes fat molecules, thereby providing additional energy in patients with severe HF and thereby preventing the wasting of lean tissue more efficiently than exogenous nutrients (47–49). On the other hand, obesity is also associated with catecholamine resistance, which may potentially confer a protective effect (48–50).

It is known that CHF patients had a high risk of experiencing malnutrition due to an imbalance that exists between the processes of protein catabolism and anabolism (51). Given that the recent guidelines employ a BMI <18.5 kg/m^2^ as a diagnostic indicator of malnutrition, a lower BMI can be considered a strong indicator of malnutrition and is, therefore, closely associated with being underweight (52). A *post hoc* analysis of the TOPCAT database found that patients with HFpEF with moderate to severe risk of malnutrition had a higher risk of adverse outcomes than those without risk for malnutrition (53). In addition, even after adjusting for lifestyle and clinical risk factors, the significant relationship between moderate to severe malnutrition risk and the cardiovascular adverse outcomes persisted. On the other hand, skeletal muscle wasting and reduced muscle strength due to sarcopenia ultimately lead to reduced exercise capacity and contribute to the development of frailty in affected patients (25). A study in Japan showed that a low BMI exhibited a strong association with a high prevalence of frailty, especially in the elderly population (54). Furthermore, this study also showed that underweight patients displayed frailty not only in terms of physical health but also in the aspects of oral and social well-being (54). These findings suggest that underweight patients may exhibit multiple types of frailty, which in turn can lead to reduced energy intake and an increased risk of malnutrition. Furthermore, malnutrition itself can lead to a reduction of muscle mass, which can exacerbate frailty development, creating a harmful cycle that culminates in a decline in BMI (55). Ultimately, this can result in an individual being underweight. Consequently, nearly all underweight patients are likely to experience concurrent malnutrition and frailty, with underweight itself representing an advanced stage of systemic illness (28). Data from the PURSUIT-HFpEF registry showed that being underweight was a trend in the Asian HFpEF population, and the patients experienced poor outcomes partly due to their frailty and malnutrition (28). Notably, a recent retrospective study showed that underweight and the risk of malnutrition independently predict in-hospital death in males but not in females with HF (56). This study differs from ours in terms of the study population (inpatients vs. outpatients) and the follow-up duration (in-hospital monitoring vs. long-term follow-up). Nevertheless, the results from Kwaśny et al. still suggest that malnutrition may not always be the primary factor contributing to increased in-hospital mortality in HF patients.

In addition to inadequate intake of protein and calories, malnutrition in heart failure patients may also encompass deficiencies in vitamins and minerals. Several micronutrients act as crucial cofactors in metabolic processes and play a vital role in optimizing energy efficiency and utilization (57). Some specific cardiomyopathies are identified that relate to genetic deficiencies in taurine, L-carnitine, and thiamine (58). It has also been revealed that several micronutrient deficiencies were present in the failing heart (59). Moreover, micronutrient supplementation could play an essential role in managing the catabolic state of cardiac cachexia (60). Consequently, the administration of both protein-energy malnutrition and micronutrient deficiencies should be considered a favorable choice for patients with heart failure.

### BMI and all-cause hospitalization risk

In the present study, BMI was not found to be an independent risk factor of all-cause hospitalization. Similar results were shown in a study on 2,501 CHF patients with HFpEF (61). Time to HF re-hospitalization is shorter in patients with high BMI compared with normal BMI. However, there was no significant difference observed in the time from follow-up to the first all-cause hospitalization between BMI categories. Recently, an analysis of data from the Kyoto Congestive Heart Failure registry involving 3,509 patients with acute decompensated HF also revealed that the BMI status did not appear to affect the risk of HF hospitalization (62). On the other hand, in a meta-analysis that included six studies, wherein the secondary outcomes were HF hospitalization rates during a follow-up period of 1.5 to 4.1 years, it was discovered that a BMI of ≥30 kg/m^2^ was linked with an equivalent risk of hospitalization due to heart failure as compared to the normal BMI group (13). Another retrospective study comprising 2,252 elderly patients with heart failure found that despite a lower incidence of death, higher BMI was associated with an increased risk of 30-day all-cause hospital readmission following HF hospitalization (63). The conflicting associations reported may be due to differences in the time period investigated and the characteristics of the populations studied. Moreover, symptoms of congestive HF, which are not correlated with BMI, are known to be the major cause of hospitalization (64). Similar to the obesity-mortality paradox, further research is required to comprehensively investigate the association between BMI and hospitalization among CHF patients.

### Implications and future directions

The current findings demonstrate a notable prevalence of underweight patients within the outpatient HF population in Vietnam and suggest that we should consider using BMI as a supplement to the existing heart failure risk models in risk classification, particularly in resource-constrained settings such as Vietnam and other low- and middle-income Asian countries due to its ease of measurement, requiring neither advanced laboratory tests nor exercise data. In addition, it is essential to pay careful attention to the follow-up of heart failure (HF) patients with underweight status due to their high mortality rates. On the other hand, the importance of recognizing and addressing frailty, sarcopenia, cachexia, and malnutrition in HF patients should also be acknowledged in order to improve their clinical outcomes. Particularly, identifying loss of weight over time may also be an important marker in this population as this has recently shown to be associated with increased all-cause and CVD specific mortality in a healthy elderly cohort (65). To achieve those goals, we recommend managing stable chronic HF patients in an outpatient heart failure program with the collaboration of a multidisciplinary team consisting of cardiologists, nutritionists, and geriatricians. Moreover, further studies are necessary to investigate in detail the impact of protein and vitamin deficiencies on clinical outcomes and evaluate the efficacy of nutritional and BMI-based interventional strategies for underweight HF patients.

### Limitations

The present study has several limitations. First, the data of other obesity-related anthropometric measures (waist and hip circumference, muscle mass, adiposity percentage), cardiorespiratory fitness, and formal frailty assessment were lacking. Second, specific serum laboratory tests for the diagnosis of malnutrition status were not performed. Third, relying solely on BMI is inadequate to evaluate body composition compartments and may result in an underestimation of sarcopenia and cachexia among obese patients. Fourth, even after appropriate adjustment, the presence of residual unmeasured confounding factors has the potential to impact the results. Therefore, the conclusion should be treated with caution. Finally, this is a single-center study, subject to limitations in selection and generalizability.

## Conclusion

This study results revealed that, among ambulatory outpatients with CHF, a lower BMI, particularly underweight, was associated with an increased risk of all-cause mortality. BMI should be considered for use in risk classification, and underweight patients should be managed by a team consisting of cardiologists, nutritionists, and geriatricians.

## Data availability statement

The raw data supporting the conclusions of this article will be made available by the authors, without undue reservation.

## Ethics statement

The studies involving humans were approved by Bach Mai Hospital’s ethics committee and institutional review board. The studies were conducted in accordance with the local legislation and institutional requirements. The participants provided their written informed consent to participate in this study.

## Author contributions

HN: supervision, conceptualization, methodology, investigation, writing – original draft preparation and editing, and visualization. TH: investigation, formal analysis, and writing – original draft preparation. HT: methodology, investigation, and writing – original draft preparation. DN: methodology, formal analysis, writing – original draft preparation and editing, and visualization. HP: conceptualization, methodology, investigation, and writing – reviewing and editing. PT: investigation and writing – original draft preparation. TP: investigation and writing – reviewing. TA: conceptualization, methodology, and writing-reviewing and editing. CR: conceptualization, methodology, and writing-reviewing and editing. JK: conceptualization, methodology, writing – reviewing and editing, and visualization. All authors contributed to the article and approved the submitted version.
