# Supplementary material for: Relationship between BMI and prognosis of chronic heart failure outpatients in Vietnam: a single-center study
Source: Front Nutr. 2023 Nov 30;10:1251601. doi: 10.3389/fnut.2023.1251601 (PMC10720040; doi:10.3389/fnut.2023.1251601)
Supplement: Supplementary file 1 [file Table_1.docx]

**Table S1. Sensitivity analysis: Multivariable Cox regression analysis for predictors of all-cause mortality**

| **Variables** | | **Multivariable model #1** | | | **Multivariable model #2** | | |
| --- | --- | --- | --- | --- | --- | --- | --- |
|  |  | **Adjusted HR** | **95% CI** | **p-value** | **Adjusted HR** | **95% CI** | **p-value** |
| **All-cause mortality** | | | | | | | |
| Age (increase 1 year) | | 1.09 | 1.04 - 1.13 | <0.001* | 1.09 | 1.04 - 1.14 | <0.001* |
| NYHA funtional class | I | 1.0 (Reference) |  |  | 1.0 (Reference) |  |  |
|  | II | 1.61 | 0.45 - 5.69 | 0.463 | 1.69 | 0.48 - 6.02 | 0.416 |
|  | III | 4.14 | 0.81 -21.30 | 0.089 | 3.62 | 0.70 - 18.74 | 0.124 |
| eGFR (<60 mL/min/1,73m^2^ ) | | 1.52 | 0.52 - 4.41 | 0.445 | 1.52 | 0.51 - 4.52 | 0.446 |
| HF phenotype | HFrEF (LVEF<40%) | 0.70 | 0.16 - 2.99 | 0.630 | 0.79 | 0.19 - 3.31 | 0.743 |
|  | HFmrEF (LVEF 41 - 49%) | 0.96 | 0.22 - 4.26 | 0.955 | 1.07 | 0.23 - 5.05 | 0.929 |
|  | HFpEF (LVEF≥50%) | 1.0 (Reference) |  |  | 1.0 (Reference) |  |  |
| BMI (decrease 1 kg/m^2^) | | 1.28 | 1.05 - 1.57 | 0.014* | - | - | - |
| BMI categories | Underweight  (BMI <18.5 kg/m^2^) | - | - | - | 3.01 | 1.06 - 8.53 | 0.039* |
|  | Normal range  (BMI 18.5–22.9 kg/m^2^) | - | - | - | 1.0 (Reference) |  |  |
|  | Overweight/Obesity  (BMI ≥23.0 kg/m^2^) | - | - | - | 0.19 | 0.02 - 1.76 | 0.145 |

Multivariable model #1: BMI as a continuous variable. Multivariable model #2: BMI as a categorial variable.

HR: Hazard ratio; 95% CI: 95% Confidence interval; NYHA: New York Heart Association; LVEF: Left ventricular ejection fraction; eGFR: estimated Glomerular filtration rate; HF: Heart failure; HFrEF: HF with reduced LVEF; HFmrEF: HF with mildly reduced LVEF; HFpEF: HF with preserved LVEF; BMI: Body mass index. *: p value <0.05.
